# Ultrastructural changes in the sublingual salivary gland of prenatal buffalo (*Bubalus bubalis*)

**DOI:** 10.14202/vetworld.2016.326-329

**Published:** 2016-03-28

**Authors:** A. D. Singh, Opinder Singh

**Affiliations:** Department of Veterinary Anatomy, Guru Angad Dev Veterinary and Animal Sciences University, Ludhiana - 141 004, Punjab, India

**Keywords:** buffalo, prenatal, sublingual salivary gland, transmission electron microscopy

## Abstract

**Aim:**

The present study was aimed to elucidate ultrastructural changes in the development of sublingual salivary gland of buffalo during prenatal life.

**Materials and Methods:**

The study was carried out on sublingual salivary gland of 36 buffalo fetuses ranging from 13.2 cm curved crown-rump length (CVRL) (88^th^ day) to full term. The fetuses were categorized into three groups based on their CVRL.

**Results:**

The cells lining the terminal tubules were undifferentiated with poorly developed cytoplasmic organelles but lacked secretory granules (SGs) at 13.2 cm CVRL (88^th^ day). The SGs appeared first in the form of membrane-bound secretory vesicles with homogeneous electron-dense as well as electron-lucent contents at 21.2 cm CVRL (122^nd^ day); however, mucous acinar cells contained electron-lucent granules, while serous secretory cells as well as serous demilunes showed electron-dense granules at 34 cm CVRL (150^th^ day) of prenatal life. At 53.5 cm CVRL (194^th^ day), both mucous and serous acini were differentiated by the density of SGs.

**Conclusion:**

The cytoplasm of acinar cells was filled with mitochondria, rough endoplasmic reticulum, and Golgi profiles in mid and late fetal age groups. The SGs were increased in number during the late fetal age group. The myoepithelial cells (MECs) were located at the base of the acinar cells as well as intercalated and striated ducts and were stellate in shape. The ultrastructure of MEC revealed a parallel stream of myofilaments in the cytoplasm and its processes. The mucous cells were predominantly present in the sublingual salivary gland and were pyramidal in shape.

## Introduction

The sublingual salivary gland contributes to the secretion of saliva which plays a key role in maintaining ruminants healthy by facilitating mastication and deglutition, helping in the restoration of normal ruminal pH. Ruminant saliva is an isotonic, bicarbonate-phosphate buffer with high pH. It has an important role in providing lubrication for eating and vocalization, aid digestion, and supply saliva for pH buffering [1]. This well-buffered solution is necessary for neutralizing acids formed by fermentation in the rumen to maintain the acid-base equilibrium of the ruminal contents. Salivary glands are composed of specialized epithelial cells, and the basic secretory units of salivary glands are clusters of cells called acini. These cells can be classified as serous cells as well as mucous cells [2]. The distribution of these types varies from species to species [3]. The salivary glands also secrete immunoglobulin A, potassium, and sodium [4].

This paucity and the precious role of the sublingual salivary gland in digestion prompted to study the histogenesis which may be serving as a tool for future research on stem cell analysis of primordia of the salivary gland. The study of prenatal development is prerequisite to understand the normal developmental biology of an organ. The documentation of normal fetal growth can serve as a guide for understanding the consequence of harmful influences at various stages of gestation [5].

Various prenatal as well as postnatal studies have been done on salivary glands of rat [6], Japanese quail [7], birds [8], and buffalo [9,10]; however, there is no detailed information about the ultrastructural studies of buffalo sublingual salivary gland during prenatal development; therefore, the present work was aimed to observe the ultrastructural changes in the sublingual salivary gland of prenatal buffalo.

## Materials and Methods

### Ethical approval

This study was conducted after approval by the Research Committee and Institutional Animal Ethics Committee.

### Collection of samples

The present study was conducted on sublingual salivary gland of 36 buffalo fetuses, during different stages of prenatal development. Immediately after collection, the fetuses were measured for their curved crown-rump length (CVRL) in centimeters with a calibrated inelastic thread. The approximate age of fetuses was calculated using the following formula in buffalo [11].

Y = 28.66 + 4.496X (CVRL <20 cm)

Y = 73.544 + 2.256X (CVRL ≥20 cm)

Where, Y is age in day(s) and X is curved crown-rump length (CVRL) in cm(s). Depending on CVRL, fetuses were divided into three groups with a minimum of 12 samples in each group:

Group I: CVRL between 0 and 20 cm
Group II: CVRL >20-40 cm
Group III: CVRL >40 cm

### Fixation and processing of samples

Immediately after measuring CVRL, the tissue samples, collected from the sublingual salivary gland of buffalo fetuses, were thoroughly washed in phosphate buffer saline solution (pH 7.4) and trimmed to 1 mm^3^ size. These samples were fixed in Karnovsky’s fixative (2.5% glutaraldehyde and 2% paraformaldehyde in 0.1 M phosphate buffer solution) for 8-12 h, and their secondary fixation was done in 2% osmium tetroxide for 2 h. Subsequently, tissue samples were dehydrated, cleared, infiltrated, embedded, and polymerized. The ultrathin sections of 70-90 nm were cut and stained with uranyl acetate for 15 min followed by lead citrate for 10 min [12]. The grids with sections were examined under transmission electron microscope for detailed study.

## Results and Discussion

### Group I

The cells lining the terminal tubules were undifferentiated with poorly developed cytoplasmic organelles but lacked secretory granules (SGs) at 13.2 cm CVRL (88^th^ day). However, the apical ends of cells showed electron-lucent and some electron-dense granules. In sublingual salivary gland of rat, at 19 days of intrauterine life, more cells of the terminal buds contained granules, and larger numbers of granules were present in the cells. Cells containing electron-lucent mucous granules and cells containing electron-dense serous granules were present around a common lumen. The mucous granules usually had fine fibrillar content; their membranes were indistinct, and adjacent granules often were fused. The size and density of serous granules varied from granule to granule, although the content of individual granules was generally of uniform density [6].

### Group II

**Figure-1:**
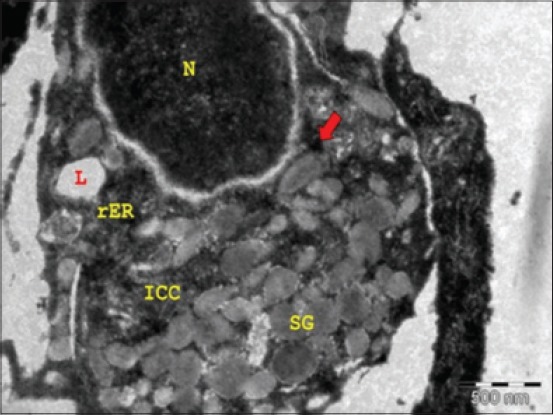
Transmission electron micrograph of sublingual salivary gland of 21.1 cm curved crown-rump length (121^st^ day) buffalo fetus showing electron-lucent secretory granules (SGs) in developing mucous acinar cells (L - Lipofuscin pigment, N - Nucleus, rER - Rough endoplasmic reticulum, ICC - Intercellular canaliculi, Arrow - Mitochondria) (×8000).

Accumulations of SGs were seen in the acinar cells at 21.1 cm CVRL (121^st^ day). Flattened myoepithelial cells (MECs) with long cytoplasmic processes containing homogeneous cytoplasm were observed around the developing acinar cells (Figure-1). These cells contained myofilaments in the cytoplasmic processes during late age groups. In sublingual salivary gland of Japanese quail, two cell types were clearly distinguished within tubules: A great majority of secretory cells with a lot of granules, and a few intercalated non-secretory cells with a high content of mitochondria. Both secretory and mitochondria-rich cells surround a lumen where secretory products are exocyted [7]. The SGs appeared in the form of membrane-bound secretory vesicles with homogeneous electron-dense as well as electron-lucent contents.

**Figure-2:**
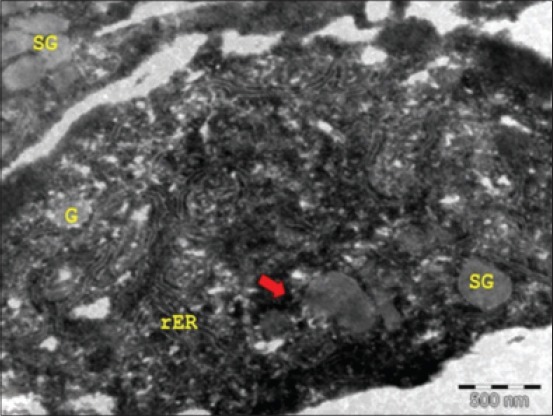
Transmission electron micrograph of the sublingual salivary gland of 34 cm curved crown-rump length (150^th^ day) buffalo fetus showing electron-lucent granules (SGs), Golgi complex (G), rough endoplasmic reticulum (rER), and mitochondria (arrow) (×8000).

At 34 cm CVRL (150^th^ day), mucous acinar cells contained electron-lucent granules, while serous cells as well as serous demilunes showed electron-dense granules. The Golgi complex, mitochondria, and rough endoplasmic reticulum (ER) were markedly developed in the cells of acini (Figure-2). The proportion of serous cells located at the periphery of the terminal buds increased from mid to late intrauterine life in the sublingual salivary gland of rat. The mixed granules had a variable density, and some contained a dense core located either peripherally or centrally within the granule [6]. The SGs might represent of storage of secretory products that are located to the heterogeneous and complex ultrastructural patterns of granules in the mucous and seromucous cells in the sublingual salivary gland of some bird species [8]. Electron-lucent granules with a delimiting membrane were observed in the trans-surface of the Golgi saccules. These observations were in accordance with the findings in buffalo [9].

### Group III

The ultrastructure of sublingual salivary gland revealed both mixed acini, mucous, and serous at 53.5 cm CVRL (194^th^ day) and was differentiated by the density of SGs. The mucous cells were predominantly present in the sublingual salivary gland and were pyramidal in shape, as reported at the 141^st^ day of prenatal life in buffalo [10]. The mucous cells had a flattened nucleus occupying the basal region of the cytoplasm and a well-organized cisternal rough ER arranged in parallel arrays. The Golgi complex was prominent and contained a significant number of smooth transport vesicles budding from the transitional ER, as well as coated vesicles formed from membranes of the Golgi saccules. In the late prenatal life of rat, the sublingual glands showed all of the epithelial structures, i.e. mucous acini, serous demilunes, intercalated ducts, striated ducts, and excretory ducts. The gland increased markedly in size, mainly through an increase in the number of acinar and demilune cells [13].

**Figure-3:**
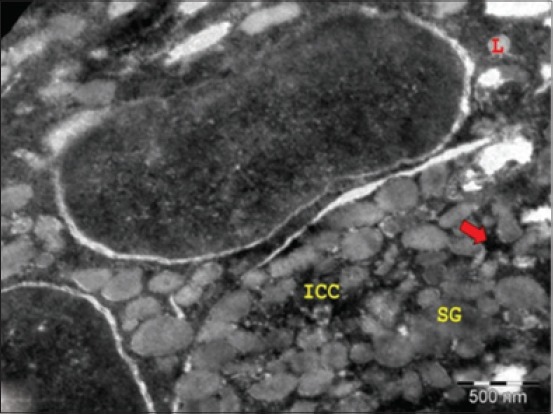
Transmission electron micrograph of sublingual salivary gland of 53.5 cm curved crown-rump length (194^st^ day) buffalo fetus showing electron-lucent secretory granules (SGs), lipofuscin pigment (L), intercellular canaliculi (ICC), and mitochondria (arrow) (×8000).

**Figure-4:**
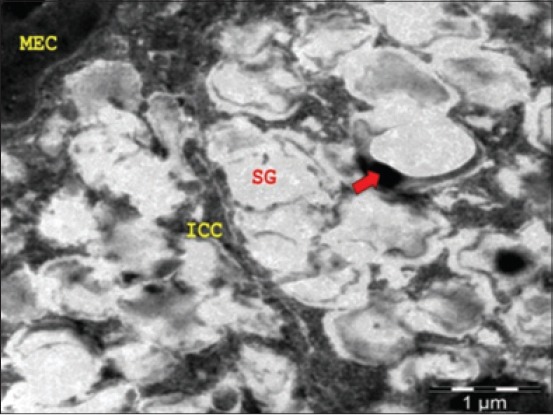
Transmission electron micrograph of sublingual salivary gland of 53.5 cm curved crown-rump length (194^th^ day) buffalo fetus showing presence of myoepithelial cell (MEC) around the basement membrane of mucous cells having electron-lucent secretory granules (SGs) (ICC - Intercellular canaliculi, Arrow - Mitochondria) (×5000).

Stellate shaped MECs with cytoplasmic processes were observed around the base of mucous acinar cells and ducts (Figure-3). These observations were in total agreement with the findings in buffalo at the 141^st^ day of prenatal life [10]. The ultrastructure of MECs revealed the parallel stream of myofilaments in the cytoplasm and its processes. These MECs were found to be attached to the glandular cells by desmosomes (Figure-4). Fat cells were observed in between the acinar cells. The size of the mucous cells and the number of granules increased during the late fetal and early postnatal period in rat. Cells containing mixed granules were present in decreasing numbers through 5 days of age [6]. There was a gradual decrease in the stromal spaces accompanied by intense collagen deposition in the intra and interlobular connective tissue. Occasionally, an electron-dense band formed by non-collagen fibrils was observed in sublingual gland close to the basement membrane of the excretory ducts. During the late intrauterine life of rat, elongated MECs were frequently seen around the acini and intercalated ducts. In contrast to the abundance of myofilaments in cell prolongation, the perinuclear cytoplasm contained a well-developed rough ER consisting of flattened and dilated cisternae, a large amount of polyribosomes and a conspicuous Golgi complex always facing the acinar or intercalated duct cells [14].

The ultrastructure of intercalated duct cells contained few electron-dense granules. The cells appeared cuboidal to low columnar with the broader base. The apical cytoplasm of the epithelial cells lining the intercalated ducts contained SGs. The basal and lateral plasma membranes of intercalated duct cells were fairly smooth with few plications, microvilli, and extensive junctional complexes. Flattened MECs with long cytoplasmic processes were observed between the epithelial cells and basement membrane of intercalated ducts. The ultrastructure of the secretory cells of rabbit sublingual gland, during last prenatal stage, was not morphologically different from that at earlier ages, except for an increase in the area of the Golgi complex in acinar cells. The larger excretory ducts were multilayered with high prismatic and basal cuboidal cells as found in adult rabbit [15].

## Conclusion

It may be concluded that the cytoplasm of acinar cells was filled with mitochondria, rough ER, and Golgi profiles in mid and late fetal age groups. The SGs appeared first in the form of membrane-bound secretory vesicles with homogeneous electron-dense as well as electron-lucent contents at 21.2 cm CVRL (122^nd^ day); however, mucous acinar cells contained electron-lucent granules, while serous secretory cells as well as serous demilunes showed electron-dense granules at 34 cm CVRL (150^th^ day) of prenatal life. The SGs were increased in number during the late fetal age group. The MECs were located at the base of the acinar cells as well as intercalated and striated ducts and were stellate in shape. The ultrastructure of MEC revealed the parallel stream of myofilaments in the cytoplasm and its processes.

## Authors’ Contributions

ADS has planned and designed the study. OS analyzed the data and provided technical support. The manuscript was prepared under the guidance of OS. All authors read and approved the final manuscript.
